# Pornography Use among Lebanese Adults: Association with Loneliness and Fear of Commitment

**DOI:** 10.3390/healthcare11060795

**Published:** 2023-03-08

**Authors:** Diana Malaeb, Souheil Hallit, Sahar Obeid

**Affiliations:** 1 College of Pharmacy, Gulf Medical University, Ajman P.O. Box 4184, United Arab Emirates; 2 School of Medicine and Medical Sciences, Holy Spirit University of Kaslik, Jounieh P.O. Box 446, Lebanon; 3 Applied Science Research Center, Applied Science Private University, Amman 11931, Jordan; 4 Research Department, Psychiatric Hospital of the Cross, Jal Eddib P.O. Box 60096, Lebanon; 5 Department of Social and Education Sciences, School of Arts and Sciences, Lebanese American University, Byblos 4504, Lebanon

**Keywords:** internet pornography use, loneliness, fear of commitment, Lebanon, adults

## Abstract

(1) Background: Sexuality is a prohibited topic about which knowledge is highly lacking among Arabs compared to Westerners, due to religious restrictions. A majority of people believes that any use of pornography is a toxic conduct that will have negative repercussions; these beliefs only shame those who engage in this behavior. Consumption of pornography can be attributed to self-reported reasons such as loneliness and fear of commitment. To date, there has been a dearth of research in Lebanon concerning pornography use. Hence, this study aims to assess the correlation between loneliness, fear of commitment, and internet pornography use among Lebanese adults. (2) Methods: This cross-sectional study conducted between October and November 2020 assessed pornography use through the Cyber-Pornography Use Inventory, Jong Gierveld Loneliness Scale, and Fear of commitment scale. It enrolled 653 adults from all Lebanese geographic areas. (3) Results: Women compared to men and Muslims compared to Christians were significantly associated with lower pornography addictive patterns scores, whereas more fear of commitment was significantly associated with higher pornography addictive patterns scores. More fear of commitment and more loneliness were significantly associated with higher having guilt regarding online porn use scores. Muslims compared to Christians had significantly lower online sexual behaviors scores. (4) Conclusions: Further research is recommended to properly assess internet pornography use and develop appropriate treatment options.

## 1. Introduction

Initially consumed through magazines, the patterns of pornography use have changed over the years so that the internet has become the primary means of consuming pornography today [1]. This shift has made pornography more accessible due to the anonymity, accessibility, and affordability that the Internet offers to the consumer [2,3]. As a result, pornography has become a normative and accepted part of our modern culture. In addition, the development of smartphones and their frequent use by young adults [4] have promoted easy access to pornography [5,6]. Online sexual behavior ranges from talking about sex with someone online to cybersex [7], which consists of online interaction for the purpose of attaining sexual stimulation [8]. Pornography refers to sexually open material that is primarily intended to sexually arouse the viewer. It has also been defined as sexually explicit material that “depicts sexual activity in an open manner, often with close-ups of the (aroused) genitals and oral, anal or vaginal penetration” [9].

The consequences of pornography use on the consumer oscillate between positive [10,11] and negative outcomes [12]. Previous findings have shown that this use opens the door to sexual autonomy and empowerment [13]. However, other studies focus on increased participation in risky sexual behaviors, such as increased number of sexual partners, sexual permissiveness, extramarital sex, and payment for sex [14,15]. Likewise, viewing pornography has a negative effect on relationships as well as sexual satisfaction and intimacy in heterosexual relationships, whether dating or conjugal, especially when the man is the pornography user [10], just as is associated with binge drinking behaviors and drug use [16].

Reports of pornography use suggest values ranging from 19% to 78.4% in women and 40% to 79% in men [17,18]. Other studies indicate that 46% to 74% of men and 16% to 41% of women are active users of pornography in modern countries [19,20]. These data are backed by one of the most widespread pornographic websites, PornHub, which reported over 39 billion searches and 42 billion visits in 2019, which suggests 115 million visits and 18,073 terabytes of data transferred per day [21]. Additionally, internet pornography use seems to be more common among young and well-educated single men [22]. Furthermore, a recent large cross-sectional online survey in Arab countries (15,027 participants) revealed that frequent pornography viewing was correlated with male gender and age group ≤15 years [23]. As for religion, individuals who ‘‘follow’’ religious values may avoid using internet pornography. In fact, research showed religiosity to negatively correlate with pornography use [24]. Moreover, lower income, less education, and longer working hours were associated with higher likelihoods of using internet pornography [25].

This enormous consumption of pornography can be attributed to having pleasure as part of masturbation [26]. However, the frequent consumption of pornography can be motivated by several factors [27]. On one hand, individual difference variables have been recognized and have emerged as motivators of pornography consumption, including, among others, personality traits [28], such as sensation seeking [29], dispositional sexual affect (erotophobia–erotophilia) [30], and narcissistic traits [31]. On the other hand, self-reported reasons are also factors favoring pornography use. Studies have reported that sexual arousal and sexual enhancement were the predominant motivations for pornography consumption among the self-reported reasons [32,33]. Besides sexual arousal and enhancement, coping and boredom [34,35], loneliness [36], as well as fear of commitment [37] are also linked to increased porn use. These last two variables will be taken into account in our study.

In the context of these two variables, a previous study in Lebanon showed a positive correlation between loneliness and social media use. According to this statement, Lebanese adults’ emotional isolation may be a major reason for their reliance on social media as their main form of communication [38,39]. On the other hand, another study revealed that financial problems are particularly challenging for Lebanese adults, which are related to their fear of emotional commitment and financial dependence [40]. Furthermore, close relationships between children and parents throughout adolescence and adulthood, as well the financial dependence among the family members, probably add to gamophobia.

According to the literature, loneliness is not due to excessive use of the internet; indeed, lonely subjects use the internet as an attempt to resolve their state of isolation [41]. Furthermore, research has shown that feelings of loneliness among adults are linked to internet pornography use [42] and cybersex [43]. As for the fear of commitment, increased pornography use was associated with lower commitment to one’s partner and higher infidelity rates [37].

Sexuality is a little discussed subject in Lebanon and this withdrawal of human sexuality from open discussion has given it a bad reputation. A majority of people believe that any use of pornography is a toxic conduct that will have negative repercussions; these beliefs only shame those who engage in this behavior [44]. Some organizations, such as some religious institutions, will condemn such behavior very explicitly [45]. Therefore, sexuality is a prohibited topic, about which knowledge is highly lacking among Arabs compared Westerners, due to religious restrictions [46]. To date, there has been a dearth of research in Lebanon concerning sexual behavior, sexual life perspectives, and quality [47]. We hypothesize that higher loneliness and fear of commitment are associated with higher internet pornography use among Lebanese adults. Hence, this study aimed to assess the correlation between loneliness, fear of commitment and internet pornography use among Lebanese adults. Therefore, the present study aimed to fill in the research gaps by answering the following research question: To what extent are loneliness and fear of commitment correlated with internet pornography use among Lebanese adults?

## 2. Materials and Methods

### 2.1. Study Design and Participants

This cross-sectional study was carried out between October and November 2020 and enrolled 653 participants. Due to the social restrictions imposed by the COVID-19 pandemic, the sample was recruited through a snowball technique from all Lebanese governorates (Beirut, Bekaa, Mount Lebanon, South Lebanon, and North Lebanon). To reach the largest possible group of subjects, participants were asked to forward the link to their contact list via social media applications such as WhatsApp, Facebook Messenger, and Instagram. Before obtaining informed consent, the participants were notified about the objective of the study and assured of the anonymity of the response. The study’s anonymity was assured to eliminate the effect of embarrassment that may be associated with pornography consumption [48]. Participants had the right to enroll in this study without any obligation or pressure from the research team, with no monetary compensation given to them for participation. The study included Lebanese people above 18 years old and who were involved in any online pornographic activity within the last 12 months. Those who have never watched porn or who were not involved in any online pornographic activity within the last 12 months were excluded from this study.

### 2.2. Ethical Aspects

The study was approved by the ethics committees of the Psychiatric Hospital of the Cross (reference: HPC-039-2020). Participants were not compensated for participation. Submitting the form online was considered equivalent to obtaining a written informed consent.

### 2.3. Minimal Sample Size 

The G-power software calculated a minimal sample of 528 participants, based on an effect size of 2% (to yield the highest minimum sample size), a 5% error, a 90% power, and 10 factors to be entered in the multivariable analysis.

### 2.4. Study Instrument and Outcomes 

Lebanese participants were asked to fill in an anonymously designed survey questionnaire in their native language (Arabic) that required approximately 30 min. Each of the participants should have completed the survey without any assistance that may influence one’s answers. The first part of the questionnaire evaluated sociodemographic information such as age, gender, religion (Christian or Muslim). The household crowding index was also calculated, dividing the number of persons living in the house by the number of rooms, excluding the bathroom and kitchen [49]. The second part of the questionnaire was composed of the different scales used: 

Cyber-Pornography Use Inventory (CPUI) is a 31-item self-report inventory composed of three subscales. Most questions involved Likert scales ranging from either strongly agree to strongly disagree (7 points) or from never to always (5 points) [50]. These subscales are as follows: (1) Pornography addictive patterns score, a fourteen-item questionnaire assessing pornography addiction in the last 12 months. Questions were evaluated through the Likert scale (Not at all/Rarely/Sometimes/Often) [16] (Cronbach’s alpha = 0.905). (2) Guilt regarding online porn use score through twelve questions addressing negative emotions, feelings, and shame following online pornography. This was evaluated through the Likert scale as follows: (Never/Rarely/Sometimes/Frequently/Always) [50] (Cronbach’s alpha = 0.863). (3) Online sexual behavior score evaluates the means of online sexual behavior using a six-item questionnaire, whether through messages, nickname, chats, sexual humor, etc. It was assessed through the following responses: Never, Rarely, Sometimes, Frequently, Always [50] (Cronbach’s alpha = 0.878).

The Jong Gierveld Loneliness Scale is a validated [38] and reliable assessment for overall, emotional, and social loneliness. It was composed of 11 items, including six negative formulations (i.e., “I miss having people around me”) and five positive formulations (i.e., “There are enough people I feel close to”), with the following three response categories: “no,” “more or less”, and “yes”. Scores for the five positively formulated items were reversed. Each item is considered as a dichotomous variable with “more or less” being merged with “no” for the positive items and with “yes” for the negative ones. The total scale score is the sum of the item scores, ranging from 0 (not lonely) to 11 (extremely lonely). A score of 3 or higher is an indication of loneliness [51] (Cronbach’s alpha = 0.735).

Fear of commitment scale is a valid and reliable measure of fear of relationship commitment that gathers social, physical, and psychological aspects related to gamophobia. The scale was composed of 17 questions following a Likert scale: 1—for strongly disagree and 4—for strongly agree. Items 1, 3, and 4 were reversed. Higher scores indicated higher fear of commitment [40] (Cronbach’s alpha = 0.851).

### 2.5. Translation Procedure

The CPUI scale was forward- and back-translated. Forward translation (English to Arabic) was performed by one translator, whereas the back translation from Arabic to English was performed by a second translator. Minor discrepancies were solved by consensus.

### 2.6. Data Analysis

Data analysis was performed on SPSS software version 25. The three scores (pornography addictive patterns score, guilt regarding online porn use, and online sexual behavior) did not follow a normal distribution; the log transformation was applied, which showed a normal distribution of the three scores. The Student’s t and ANOVA tests were used to compare two and three or more means, respectively. Pearson test was used to correlate two continuous variables. Three linear regressions were conducted, taking the pornography addictive patterns, guilt regarding online porn use, and online sexual behavior scores as dependent variables. Variables that showed a *p* < 0.25 in the bivariate analysis were entered as independent ones in the final models. A *p* < 0.05 was considered significant.

## 3. Results

### 3.1. Study Sample Characteristics

A total of 653 participants agreed to partake in this study. The mean age was 23.92 ± 5.30 years, with 70.1% females. Other descriptive details can be found in Table 1.

When dichotomizing the three scores according to their respective medians, the results showed that 375 (57.4%) participants had porn addictive patterns, 329 (50.4%) had guild regarding online porn use, whereas 189 (28.9%) had online sexual behaviors.

### 3.2. Bivariate Analysis

The results of the bivariate analysis are summarized in Table 2 and Table 3. Males had higher pornography addictive patterns. Moreover, higher loneliness was significantly associated with more guilt regarding online porn use, whereas higher fear of commitment was correlated with more pornography addictive patterns, more guilt regarding online porn use, and more online sexual behaviors.

### 3.3. Multivariable Analyses

#### 3.3.1. Pornography Addictive Patterns 

Women compared to men (Beta = −0.21) and Muslims compared to Christians (Beta = −0.12) were significantly associated with lower pornography addictive patterns scores, whereas more fear of commitment (Beta = 0.009) was significantly associated with higher pornography addictive patterns scores (Table 4, Model 1).

#### 3.3.2. Guilt Regarding Online Porn Use

More fear of commitment (Beta = 0.005) and more loneliness (Beta = 0.04) were significantly associated with higher having guilt regarding online porn use scores (Table 4, Model 2).

#### 3.3.3. Online Sexual Behaviors

Muslims compared to Christians (Beta = −0.14) had significantly lower online sexual behaviors scores (Table 4, Model 3).

## 4. Discussion

### 4.1. Loneliness

The study results revealed that mental health impairment, exhibited by loneliness, was associated with higher guilt regarding porn use and online sexual behavior; even though the association between pornography consumption and mental and physical health remains ambiguous [52,53,54]. The findings are compatible with stress perceived, enacted stigma, lack of social support, and loss of friends that generate loneliness [55], leading to the use of sex as a coping strategy to fight isolation [56]. Furthermore, Fonagy et al. [57] (p. 794) note that oxytocin—released during the sexual response cycle—can “inhibit the neural systems that underpin the generation of negative affect”. Pornography and sexual arousal may therefore be used as a temporary escape from upsetting loneliness [58]. The potential for compulsivity or addiction to the sexual response cycle being exploited autoerotically, outside the grounding context of the requirements of a pair-bond relationship, is suggested by the link between the brain reward system stimulated by the sexual response cycle and the addiction reward circuit.

### 4.2. Fear of Commitment

Results showed that more fear of commitment was associated with higher internet pornography use. These findings may be attributable to their partners’ regular pornography use, resulting in an unstable relationship and greater fear of commitment [59]. Additionally, pornography impairs a partners’ behavior, with them tending to become dominating and less caring towards marriage relationship basis [60]. A further explanation is that pornography leads to an increased desire for various sexual activities and heightened attention to relationship alternatives, thereby restraining commitment to the primary partner [59].

### 4.3. Gender

Our results showed that females were significantly associated with lower pornography addictive patterns, lower guilt regarding online porn use, and lower online sexual behaviors, consistent with other findings [61]. Although the literature highlighted that women are at risk of developing pornography addiction, males appear to be much more prone. According to a previous study, there are differences in gender regarding pornography viewpoints since males find it more attractive than their counterparts and prefer many novel sexual partners, which is apparently the most efficient way to pass on their genetics [62]. In addition, modern public attitudes and norms towards social acceptance of male sexual behavior disseminate the preference for pornography, whereas female sexual behavior tends to be highly prohibited and therefore restrains pornography use [63]. Similarly, it seems that females’ access to pornography lacks social acceptance even in Western culture [14]. Furthermore, it has been concluded in a previous study that around 16–31% of women report using pornography regularly, whereas around 46% of males use pornography weekly [17]. Moreover, women feel ashamed to use pornography, which might explain their lower exposure.

### 4.4. Religion

The results of our study showed that Muslims had significantly lower porn addictive patterns and online sexual behavior compared to Christians. This is understandable given that previous research on attitudes toward pornography have found strong links between high religiosity, being affiliated to a conservative religion (such as Islam), and condemnation of pornography use [24,64]. In this line, engaging in pornography use in Islam may be against one’s religion, regarded as an act of lust and sin [20]. Furthermore, subjects who are religiously conservative generally favor more traditional sexual values, such as opposition to homosexuality [65], avoidance of premarital sex [66], and less acceptance of pornography use. Likely due to these conservative sexual values, and possible anxiety surrounding the use of pornography, religious individuals consistently report lower levels of pornography use than secular populations [67].

### 4.5. Clinical Implications

Our study adds useful information towards pornography use in Lebanon, compatible with international findings concerning sex in general. Moreover, it allows for implementing interventional and preventive measures to raise awareness about pornography and its associated factors (loneliness and fear of commitment). Hence, psychological and psychiatric support limit the factors that favor pornography and minimize the risk of addiction. Furthermore, this study provides much needed preliminary good practice indicators for mental health practitioners about how to engage people in conversations about their internet pornography use and respond to the issues raised. Finally, given the momentum provided by public discourse, academic research, and policy recommendations, education-based initiatives to reduce the harmful effects of pornography and factors linked to pornography among children and youth are likely to intensify in the near future [68]. Hence, education on pornography must be a part of education curricula.

### 4.6. Limitations and Strengths

While the study did consider a multitude of variables, it has several limitations. It used self-report measures, because of which some participants may have lied due to the topic’s sensitive nature [16] despite assuring them of anonymity. Some factors may have been underreported, such as personality traits that are generally seen as weaknesses in Lebanese culture. The temporal relationship could not be studied because of the cross-sectional design of the study. Thus, our study may be subject to both selection and information bias that cofound our results, although potential confounding factors were accounted for in the statistical analysis. Another limitation is the uncertainty about whether the actual pornography use or participants’ suspicion of addictive usage causes their psychological distress. Our study also used some non-validated scales, which may raise the question about the accuracy of generated results. Residual confounding bias may be possible, as not all factors related to internet pornography use were considered in our study. However, the study’s intention and value reside in the data uniqueness and richness in depth and description.

## 5. Conclusions

This study highlights the underlying issues surrounding individuals’ pornography use, which can aid in informing mental health professionals and increasing their competence to treat those who present with pornography use. Therefore, reducing symptoms of loneliness and improving commitment to relations would constitute sound targets for psychological interventions in people who are experiencing dysfunctional and impaired use of pornography or searches for online sexual contact. For future studies related to internet pornography use, it is recommended to use the online photovoice to venture out of traditional research and clinical roles, and adopt a social justice, multicultural, and contextually appropriate approach [69,70]. Such an approach enables researchers to reach out to the community in an appropriate and effective way [71] and helps them to understand a topic based on the perspectives and stories of the participants to improve their psychological state [72] and reduce the experience of stigmatization and shame [73].

## Figures and Tables

**Table 1:** Sociodemographic characteristics of the participants (N = 653).

Variable	N (%)
**Gender**	
Male	195 (29.9%)
Female	458 (70.1%)
**District**	
Beirut	104 (15.9%)
Mount Lebanon	220 (33.7%)
North Lebanon	93 (14.2%)
South Lebanon	156 (23.9%)
Bekaa	80 (12.3%)
**Education level**	
Secondary or less	115 (17.6%)
University	538 (82.4%)
**Marital status**	
Single/divorced/widowed	553 (84.7%)
Married	100 (15.3%)
**Religion**	
Christian	68 (10.4%)
Muslim/Druze	585 (89.6%)
	**Mean ± SD**
**Age (in years)**	23.92 ± 5.30
**Household crowding index**	1.12 ± 0.60
**Pornography addictive patterns score**	8.06 ± 10.47
**Guilt regarding online porn use score**	5.10 ± 6.79
**Online sexual behavior score**	1.43 ± 3.00

**Table 2:** Bivariate analysis of categorical variables associated with the three pornography scores.

Variable	Pornography Addictive Patterns	*p*	Guilt Regarding Online Porn Use	*p*	Online Sexual Behavior	*p*
**Gender**		<0.001		0.471		0.487
Men	1.06 ± 0.40		0.92 ± 0.33		0.59 ± 0.32	
Women	0.86 ± 0.37		0.89 ± 0.34		0.55 ± 0.37	
**Marital status**		0.368		0.083		0.471
Single/divorced/widowed	0.94 ± 0.40		0.91 ± 0.33		0.58 ± 0.35	
Married	0.89 ± 0.35		0.81 ± 0.34		0.52 ± 0.32	
**Religion**		0.065		0.339		0.058
Christian	1.03 ± 0.39		0.85 ± 0.38		0.68 ± 0.37	
Muslim/Druze	0.92 ± 0.39		0.91 ± 0.33		0.55 ± 0.34	
**Education level**		0.929		0.830		0.113
Secondary or less	0.93 ± 0.43		0.89 ± 0.35		0.66 ± 0.33	
University	0.93 ± 0.39		0.90 ± 0.33		0.55 ± 0.35	

The log scores were used in this analysis.

**Table 3:** Correlation matrix of the continuous scores.

	1	2	3	4	5	6	7
1. Porn addictive patterns	1						
2. Guilt regarding online porn use	0.46 ***	1					
3. Online sexual behavior	0.48 ***	0.54 ***	1				
4. Age	0.06	−0.07	0.08	1			
5. Household crowding index	0.02	−0.09	−0.02	−0.04	1		
6. Loneliness	0.09	0.24 ***	0.13	−0.05	0.02	1	
7. Fear of commitment	0.16 **	0.19 **	0.16 *	−0.14 ***	−0.02	0.29 ***	1

* *p* < 0.05; ** *p* < 0.01; *** *p* < 0.001; the log scores were used in this analysis.

**Table 4:** Linear regressions models taking the porn addictive patterns, guilt regarding online porn use, and online sexual behavior scores as dependent variables.

Variable	Unstandardized Beta	Standardized Beta	*p*	95% CI
**Model 1: Porn addictive patterns score as the dependent variable**				
Gender (women vs. men *)	−0.21	−0.25	<0.001	−0.28; −0.13
Religion (Muslim vs. Christian *)	−0.12	−0.10	0.037	−0.23; −0.01
Age	0.01	0.07	0.124	−0.001; 0.01
Loneliness	0.01	0.04	0.368	−0.01; 0.03
Fear of commitment	0.009	0.18	<0.001	0.004; 0.014
**Model 2: Guilt regarding online porn use score as the dependent variable**				
Age	0.001	0.004	0.953	−0.01; 0.01
Loneliness	0.04	0.19	0.001	0.02; 0.07
Fear of commitment	0.005	0.13	0.028	0.001; 0.010
Marital status (married vs. single *)	−0.10	−0.10	0.183	−0.24; 0.05
Household crowding index	−0.05	−0.09	0.097	−0.10; 0.01
**Model 3: Online sexual behavior score as the dependent variable**				
Loneliness	0.02	0.10	0.162	−0.01; 0.05
Fear of commitment	0.01	0.13	0.076	−0.001; 0.01
Religion (Muslim vs. Christian *)	−0.14	−0.14	0.049	−0.28; −0.001
Education (university vs. secondary or less *)	−0.07	−0.07	0.308	−0.21; 0.07

* Reference group; The log scores were used in this analysis; numbers in bold indicate significant *p* values.

## Data Availability

The authors do not have the right to share any data information as per the ethics committee rules and regulations.
